# Mechanism- and Immune Landscape-Based Ranking of Therapeutic Responsiveness of 22 Major Human Cancers to Next Generation Anti-CTLA-4 Antibodies

**DOI:** 10.3390/cancers12020284

**Published:** 2020-01-24

**Authors:** Peng Zhang, Xinxin Xiong, Christian Rolfo, Xuexiang Du, Yan Zhang, Han Yang, Alessandro Russo, Martin Devenport, Penghui Zhou, Yang Liu, Pan Zheng

**Affiliations:** 1 Division of Immunotherapy, Institute of Human Virology and Department of Surgery, University of Maryland Baltimore School of Medicine, Baltimore, MD 21201, USA; peng.zhang@ihv.umaryland.edu (P.Z.); Xuexiang.Du@ihv.umaryland.edu (X.D.); Yan.Zhang@ihv.umaryland.edu (Y.Z.); 2 Key Laboratory of Oncology in Southern China, Collaborative Innovation Center for Cancer Medicine, Sun Yat-Sen University Cancer Center, Guangzhou 510060, China; xiongxx@sysucc.org.cn (X.X.); yanghan@sysucc.org.cn (H.Y.); zhouph@sysucc.org.cn (P.Z.); 3 Greenebaum Comprehensive Cancer Center, Experimental Therapeutics Program, University of Maryland Baltimore School of Medicine, Baltimore, MD 21201, USA; alessandro-russo@alice.it; 4 Medical Oncology Unit, A.O. Papardo & Department of Human Pathology, University of Messina, 98100 Messina, Italy; 5 OncoImmune, Inc.Rockville, MD 20850, USA; mdevenport@oncoimmune.com

**Keywords:** Anti-CTLA-4 antibody, lung cancer, immunotherapy responsiveness, TCGA, treg, ADCC/ADCP, irAE

## Abstract

Background: CTLA-4 was the first immune checkpoint targeted for cancer therapy and the first target validated by the FDA (Food and Drug Administration) after approval of the anti-CTLA-4 antibody, Ipilimumab. However, clinical response rates to anti-CTLA-4 antibodies are lower while the rates of immunotherapy-related adverse events (irAE) are higher than with anti-PD-1 antibodies. As a result, the effort to target CTLA-4 for cancer immunotherapy has stagnated. To reinvigorate CTLA-4-targeted immunotherapy, we and others have reported that rather than blocking CTLA-4 interaction with its cognate targets, CD80 and CD86, anti-CTLA-4 antibodies achieve their therapeutic responses through selective depletion of regulatory T cells in the tumor microenvironment. Accordingly, we have developed a new generation of anti-CTLA-4 antibodies with reduced irAE and enhanced antibody-dependent cell-mediated cytotoxicity/phagocytosis (ADCC/ADCP). A major unresolved issue is how to select appropriate cancer types for future clinical development. Methods: We generated a landscape of the immune tumor microenvironment from RNAseq and genomic data of 7279 independent cancer samples belonging to 22 cancer types from The Cancer Genomics Atlas (TCGA) database. Based primarily on genomic and RNAseq data from pre-treatment clinical samples of melanoma patients who were later identified as responders and nonresponders to the anti-CTLA-4 antibody Ipilimumab, we identified 5 ranking components of responsiveness to anti-CTLA-4, including CTLA-4 gene expression, ADCC potential, mutation burden, as well as gene enrichment and cellular composition that favor CTLA-4 responsiveness. The total ranking number was calculated by the sum of 5 independent partitioning values, each comprised of 1–3 components. Results: Our analyses predict metastatic melanoma as the most responsive cancer, as expected. Surprisingly, non-small cell lung carcinoma (NSCLC) is predicted to be highly responsive to anti-CTLA-4 antibodies. Single-cell RNAseq analysis and flow cytometry of human NSCLC-infiltrating T cells supports the potential of anti-CTLA-4 antibodies to selectively deplete intratumoral Treg. Conclusions: Our in silico and experimental analyses suggest that non-small cell lung carcinoma will likely respond to a new generation of anti-CTLA-4 monoclonal antibodies. Our approach provides an objective ranking of the sensitivity of human cancers to anti-CTLA-4 antibodies. The comprehensive ranking of major cancer types provides a roadmap for clinical development of the next generation of anti-CTLA-4 antibodies.

## 1. Background

As the first immune checkpoint explored for cancer immunotherapy, CTLA-4 was validated as an immunotherapeutic target after FDA approval of Ipilimumab for human use, either as monotherapy for melanoma, or as part of combination therapy with the anti-PD-1 antibody, Nivolumab, in melanoma, renal cancer, and colorectal cancer with microsatellite instability [1,2,3,4,5]. However, while clinical trials using antibodies targeting PD-1 and its ligand B7-H1/PD-L1 have resulted in the regulatory approval for use of multiple antibodies for multiple cancer types, clinical trials with Ipilimumab have met high profile failures in multiple phase III clinical trials, due to considerably higher toxicity, including fatal ones [6,7,8]. Moreover, randomized and controlled studies using Tremelimumab, the other anti-CTLA-4 antibody with extensive clinical experience either as monotherapy or combination therapy with an anti-PD-L1 reagent, also resulted in multiple failures in phase IIb and III clinical trials [9,10]. Therefore, new approaches are needed for the clinical development of therapeutics targeting CTLA-4.

An important development in cancer immunotherapy is a re-evaluation of the mechanism by which anti-CTLA-4 antibodies induce tumor rejection [11,12]. First, we have reported that blocking the interaction between CTLA-4 and its cognate ligand CD80 and CD86 is neither necessary nor sufficient for anti-CTLA-4-induced tumor rejection [13]. Second, several groups, including ours [13,14,15,16,17], have reported that the therapeutic effect of anti-CTLA-4 antibodies requires ADCC activity that selectively depletes regulatory T cells in the tumor microenvironment. These two lines of fundamental studies have inspired the development of the next generation of anti-CTLA-4 antibodies with enhanced ADCC activity or preferential activation in the tumor microenvironment [18,19]. Preclinical studies have revealed that this new generation of antibodies exhibit more potent anti-tumor effects in vivo when tested in mice with large established tumors [19].

Another challenge in CTLA-4 targeting are the severe adverse effects associated with the clinically used anti-CTLA-4 called immunotherapy-related adverse events (irAE) [20,21,22]. This toxicity limits the dose and frequency of the administration of anti-CTLA-4 antibodies. Notably, enhancing ADCC activity of Ipilimumab through the production of afucosylated form appears to have significantly increased toxicity in a non-human primates [23]. In order to understand the mechanism of irAE, we have developed a novel human CTLA-4 knockin mice model that fully recapitulates clinical irAEs [18]. Using this model, we were able not only to uncouple irAEs from the cancer immunotherapeutic effect (CITE), but also demonstrate that irAE can be avoided by preventing anti-CTLA-4 antibody-induced lysosomal degradation of the CTLA-4 molecules [18,19]. This is achieved by using pH-sensitive antibodies that dissociate from CTLA-4 prior to lysosomal degradation and allow its recycling. Unlike afucosylated anti-CTLA-4 antibodies, the pH-sensitive antibodies not only exhibit stronger ADCC and thus more efficient intratumor Treg depletion, but also preserves the physiological function of CTLA-4 to avoid irAE. This new generation of anti-CTLA-4 antibodies will likely lead to a new wave of clinical studies testing CTLA-4 targeting for cancer immunotherapy.

Given the large number of failures in previous trials, an important issue is which cancers are suitable indications for the next generation of anti-CTLA-4 antibodies. Here we develop a strategy that integrates previous experience with Ipilimumab with genomic and expression profiles of major human cancers in order to rank cancer indications for their likely response to the next generation of anti-CTLA-4 antibodies. We mined the anti-CTLA-4 immune response dataset consisting of pre-treated melanoma samples from responders and non-responders to Ipilimumab for gene signatures pertaining to objective tumor response. Based on a new mechanism of action, we performed an in-depth survey of the TCGA database for genomic and RNA expression features that promote anti-CTLA-4 response, including target (CTLA-4) expression, gene signature, immune cell composition, tumor mutation burden, and ADCC/ADCP features. 

## 2. Methods

### 2.1. Clinical Patient Samples and Database

We collected a total of 7279 samples from 22 cancer types (LUAD: Lung adenocarcinoma, *n* = 493; LUSC: Lung squamous cell carcinoma, *n =* 494; SKCM-TM: Skin Cutaneous Melanoma—metastasis, *n =* 351; SKCMTP: Skin Cutaneous Melanoma—primary, *n =* 101; HNSC: Head and Neck squamous cell carcinoma, *n =* 498; BRCA-Basal: Breast invasive carcinoma—basal, *n =* 169; BRCAHer2: Breast invasive carcinoma—Her2, *n =* 78; BRCA-LumB: Breast invasive carcinoma—LumB, *n =* 189; BRCA-LumA: Breast invasive carcinoma—LumA, *n =* 492; STAD: Stomach adenocarcinoma, *n =* 368; ESCA: Esophageal Carcinoma, *n* = 161; PAAD: Pancreatic adenocarcinoma, *n* = 177; COAD: Colon adenocarcinoma, *n* = 439; READ: Rectum adenocarcinoma, *n* = 163; BLCA: Bladder Urothelial Carcinoma, *n* = 402; KIRC: Kidney renal clear cell carcinoma, *n* = 526; KIRP: Kidney renal papillary cell carcinoma, *n* = 287; PRAD: Prostate adenocarcinoma, *n* = 492; OV: Ovarian serous cystadenocarcinoma, *n* = 370; LIHC: Liver hepatocellular carcinoma, *n* = 370; GBM: Glioblastoma multiforme, *n* = 150; LGG: Brain Lower Grade Glioma, *n* = 509) from the largest publicly available cancer genomics database, namely The Cancer Genome Atlas (TCGA) with genomic, transcriptomic and clinical data. We accessed the TCGA data portal [24] and downloaded mRNA expression quantification profiles (HTSeq–FPKM). Genomic profiles (somatic mutation and germline SNP) and clinical data files of cancer samples were downloaded from cBioPortal for Cancer Genomics [25]. The immunological data files and annotated signature files of cancer samples were downloaded from the NIH Genomic Data Commons [26]. And we used the CIBERSORT [27] framework to comprehensively estimate the cell abundance of infiltrated immune cells within the tumor microenvironment.

### 2.2. Studies with Human Samples

Tissue biopsy samples from 9 consented patients (Supplementary Materials Table S1) with NSCLC from the SunYat—sen University Cancer Center were used for this study after approval by the SunYat-sen University Cancer Center Research Ethics Board (IRB No. SZR2019-016). Written informed consent for publication of their clinical details was obtained from each patients or a relative of the patient.

Tumor tissue was dissected into fragments (approximately 1–2 mm^3^) using a sterile scalpel in petri dishes containing 10 mL of PBS containing 2% fetal bovine serum, and tissue fragments of the cell suspension were washed two times with ice-cold PBS. Tumor tissues were then re-suspended in 15 mL RPMI supplemented with 2% FBS, 50U/mL Collagenase Type IV (Invitrogen), 20U/mL DNase (Roche) and incubated at 37 °C for 2 h, and tissue was further dissociated using a gentleMACS Dissociator (Miltenyi Biotech). Suspensions were washed three times with PBS and passed through a 70 mM strainer. All the antibodies were purchased from Biolegend or BD Biosciences. Cells were washed once in 100 μL of PBS containing 2% fetal bovine serum and labeled on ice. For surface marker staining, 2 million cells were suspended in 200 μL PBS and stained with 1 μL of surface Abs at 4 °C for 30 min in the dark, followed by washing twice with 1 mL PBS containing 2% fetal bovine serum. The cell pellet was resuspended with 500 uL 1× Fix buffer (BD Biosciences) and kept in the dark at 4 °C for 30 min. 500 µL 1× permeabilization wash buffer (BD Biosciences) was added to the tube and centrifuged at 1200 rpm for 5 min. Supernatant was discarded carefully without disturbing cells on the bottom and the cell pellet resuspended with 100 µL of 1× permeabilization wash buffer. Then 1 µL antibody was added for intracellular staining and incubated for 45 min at 4 °C in the dark. The cells were then washed twice with permeabilization wash buffer and re-suspended in 500 µL PBS containing 2% fetal bovine serum. Flow cytometry was performed on a BD Fortesa ×20 flow cytometer, and results were analyzed using the FlowJo software.

### 2.3. Experimental Animals

C57BL/6 mice that express the CTLA-4 protein with 100% identity to human CTLA-4 protein under the control of the endogenous mouse Ctla4 locus have been previously described [28]. All mice were maintained at the Research Animal Facility of the Institute of Human Virology at the University of Maryland Baltimore School of Medicine. All studies involving mice were approved by the Institutional Animal Care and Use Committee (IACUC, Protocol No. 0218011).

### 2.4. Antibodies

The humanized anti-CTLA-4 mAb, HL32, has been previously described [13,18,19] and was produced by Sydlabs, Inc. (Boston, MA, USA). Recombinant Ipilimumab with the amino acid sequence disclosed in WC500109302 and http://www.drugbank.ca/drugs/DB06186 was produced by Sydlab, Inc. (Boston, MA, USA). Azide-free human IgG-Fc was purchased from Athens Research and Technology (Athens, GA, USA).

### 2.5. Flow Cytometry for Mouse Samples

Cells were stained with fluorochrome-conjugated monoclonal antibodies against mouse CD8 (Cat# 100748, Biolegend, Cat# 45-0081-82, eBioscience), mouse CD4 (Cat# 100453, Biolegend, Cat# 100531, Biolegend), mouse CD45 (Cat# 103151, Biolegend, Cat# 47-0451-82, eBioscience), mouse Foxp3 (Cat# 48-5773-82, Thermo Fisher Scientific, Cat# 17-5773-82, eBioscience), human CTLA-4 (Cat# 369604, Biolegend), and Mouse IgG2a, κ Isotype Ctrl CTLA-4 (Cat# 400214, Biolegend). All the samples were stained with LIVE/DEAD^®^ Fixable Aqua Dead Cell Stain Kit (Cat# L34957, Thermo Fisher Scientific) to exclude the dead cells. Intracellular staining was performed with the Intracellular Fixation and Permeabilization kit (Cat# 88-8824, eBioscience) according to the manufacturer’s instructions. The samples were analyzed by the BD Canton II or Cytek ™ Aurora Flow cytometer and data were analyzed by Flowjo software.

### 2.6. Gene-Set Enrichment Analysis

Gene-set enrichment analysis was performed with the GSEA [29] program (v. 3.0). The Broad Molecular Signatures Database (MSigDB v6.0) C2: curated gene sets (KEGG pathway) was used, which summarize and represent specific well-defined biological states or processes. The GSEA program was run with 1000 permutations for statistical significance estimation, and the default signal-to-noise metric between the two phenotypes was used to rank all genes. Single sample gene-set enrichment analysis (ssGSEA) was implemented for the immune signature score estimation by using “GSVA” R package.

### 2.7. Ranking Cancer Types for Their Response to Anti-CTLA-4

The total ranking number was calculated by the sum of 5 independent partitioning values. We used the following formula to estimate the total ranking number for each cancer type:

$$RANK_{total}=\left(\overset{3}{\underset{i=1}{\sum }}RANK_{ADCC}\right)/3+\;RANK_{CTLA4exp}+\left(\overset{3}{\underset{i=1}{\sum }}RANK_{signature_{i}}\right)/3\;+\left(\overset{3}{\underset{i=1}{\sum }}RANK_{ImmCell_{i}}\right)/3+\left(\overset{2}{\underset{i=1}{\sum }}RANK_{MutBurden_{i}}\right)/2$$

where the *RANK_ADCC_* represents the “ADCC Features” partitioning, that was the mean value of 3 ranking numbers based on the estimated cell fraction of Treg cells, *FCGR3A* gene expression, and *FCGR3A* SNP rate for V158F.

*RANK_CTLA_*_4*exp*_ represents the “CTLA4 Expression” partitioning, that was the ranking number based on the mean value of CTLA-4 expression of all cancer types.

*RANK_signature_* represents the “Signature Genes Score” partitioning, that was the mean value of ranking numbers based on the 3 immunological signature scores (“Positive regulation of Adaptive Immune Response”, “Lymphocyte Activation” and “CD8 T cell effector markers”).

*RANK_ImmCell_* represents the “Immune Cell Infiltration” partitioning, that was the mean value of ranking numbers of CD8 T cell and Macrophage M1 cell fraction and reversed ranking number of CD4 T cell memory resting cell fraction.

*RANK_MutBurden_* represents the “Mutational Burden” partitioning, that was the mean value of ranking numbers based on the non-silent mutation rate and neo-antigen count. As the neoantigen burden data of metastasis samples of Skin Cutaneous Melanoma (SKCM) was a lack in PamImmu database, the ranking number for SKCM-metastasis was set to the rank of the non-silent mutation rate.

### 2.8. Single Cell Sequencing Data Analysis

The gene expression count profile was accessed from Gene Expression Omnibus [30]. Analyses were then performed using R software [31] primarily using the “Seurat” package. To process data, cells were filtered, keeping only those cells with the number of genes detected per cell > 300 and < 4000, and percent mitochondrial genes < 0.10. Samples were then log-normalized and scaled whereby number and two variables (unique molecular identifiers (UMIs) and percent mitochondrial genes) were regressed out. Clusters were determined using the first 10 principal components and graphed using tSNE dimensional reduction for each sample. Each cluster was defined based on clustering and marker genes.

### 2.9. Biostatistical Analysis

Data were analyzed using a Mann–Whitney test to compare between two groups and oneway analysis of variance (ANOVA) for multiple comparisons. For the differential expression analysis, the Mann–Whitney test with multiple testing adjustment (False Discovery Rate, FDR) determined the significant difference. Statistical calculations were performed using GraphPad Prism software (GraphPad Software, San Diego, CA, USA) or R Software [31]. The data from human and mouse tissues are presented as mean ± standard deviation (SD). The Student t test was used to determine the statistical significance of differences between samples. Analyses were performed using Graphpad Prism 8.0 software, * *p* < 0.05, ** *p* < 0.01, *** *p* < 0.001.

The enriched analysis of gene ontology terms for up- and down-regulated genes were performed by DAVID Functional Annotation Tool [32].

## 3. Results

### 3.1. Selective Intratumoral Treg Depletion in TUMOR-BEARING MICE—SELECTIVITY in Treg Depletion by a pH-Sensitive Anti-CTLA-4 Antibody

**Figure 1:**
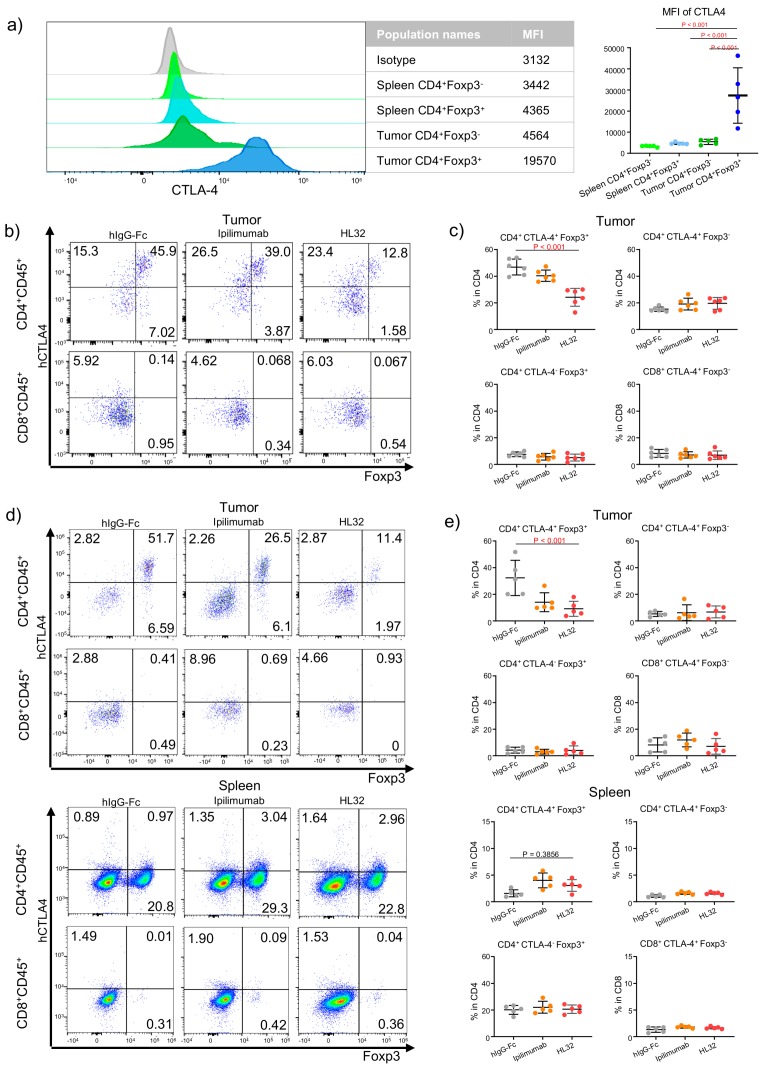
Improving Treg depletion in the tumor microenvironment without jeopardizing the selectivity of anti-CTLA-4 antibodies. (**a**) Comparing cell surface CTLA-4 levels among different T cell subsets. The left panel shows histograms depicting the distribution of cell surface CTLA-4 on gated T cell subsets. The right panel show summary data of mean fluorescence intensity of cell-surface CTLA-4 staining from 5 tumor-baring mice. 5 × 10^5^ MC38 tumor cells were injected (s.c.) into *Ctla*4*^h^*^/*h*^ mice, and mice were treated (i.p.) with 100 μg/mouse control hIgG-Fc per mouse on day 6 after tumor inoculation. Single-cell suspensions were prepared from tumor and spleen on day 10. (**b**,**c**) Selectivity of Treg depletion at 24 h post anti-CTLA-4 treatment. 5 × 10^5^ MC38 tumor cells were injected (s.c.) into *Ctla*4*^h^*^/*h*^ mice, and mice were treated (i.p.) with 100 μg Ipilimumab, HL32 or control hIgG-Fc per mouse on day 14 after tumor inoculation and single cell suspensions were analyzed for expression of CD4, CD8, total CTLA-4 and FOXP3. (**b**) Representative flow cytometry profiles depicting the proportion of different subpopulations among single-cell suspensions prepared from tumor tissues (upper panel) and spleen tissues (lower panel) on day 15. (**c**) Summary data of the percentage on different subpopulations from tumor tissues (upper panel) and spleen tissues (lower panel). The T cell subset gating strategy is based on quardrons shown in (**b**). (**d**,**e**). As in (**b**,**c**), except that tumors and spleens were harvested at 96 h post antibody treatment and that the cell surface CTLA-4 were measured. Data shown were representative of 2–3 independent experiments. Statistical significance was determined by the one-way ANOVA in panel (**c**,**e**).

We and others have previously reported that anti-CTLA-4 antibodies, including Ipilimumab selectively depleted Treg in the mouse tumor microenvironment but not in the spleen [13,16]. However, it is less clear if some CTLA-4-expressing effector T cells are subject to depletion by anti-CTLA-4 when antibodies with enhanced ADCC [19] are employed. More recently, we reported that pH-sensitive anti-CTLA-4 antibodies are more effective in ADCC and tumor rejection [19], which raise the issue as to whether increasing ADCC activity jeopardizes the selectivity of the antibodies. As shown in Figure 1a, among the tumor-infiltrating CD4 T cells, essentially all of the Foxp3^+^ population expressed high levels of cell surface CTLA-4. While most Foxp3^−^ CD4 T cells are negative for cell surface CTLA-4, a distinct population of cell surface CTLA-4^+^ cells are identifiable (Figure 1b), making them potential targets for anti-CTLA-4 antibodies. Spleen Treg expressed detectable although low levels of cell surface CTLA-4, while non-Treg CD4 T cells expressed no detectable cell surface CTLA-4 as expected (Figure 1c,d).

To test selectivity of the anti-CTLA-4 antibodies, we treated tumor bearing mice with anti- CTLA-4 antibodies, including pH-sensitive HL32 and pH-insensitive Ipilimumab. Within one day of treatment, significant Treg depletion was achieved only with pH-sensitive anti- CTLA-4 antibodies (HL32], but not Ipilimumab, as previously reported [19]. To determine selectivity, we divided the tumor-infiltrating CD4 and CD8 T cells into four subsets: CD4^+^CTLA4^+^Foxp3^+^, CD4^+^CTLA4^−^Foxp3^+^, CD4^+^CTLA4^+^Foxp3^−^, and CD8^+^CTLA4^+^Foxp3^−^ based on profiles in Figure 1b and compared their response to HL-32 and Ipilimumab. As shown in Figure 1b,c, HL32 reduced percentages of the CD4^+^CTLA4^+^Foxp3^+^ population only. Notably, no reduction of the percentages of the intratumorial CD4^+^CTLA4^+^Foxp3^−^ cells was observed even though a sizable population within this subset expressed significant levels of cell surface CTLA-4 (Figure 1a). As expected, Ipilimumab did not cause depletion in any subset at this time point.

At 96 h after antibody treatment, depletion of tumor-infiltrating Treg is observed in mice receiving either Ipilimumab or HL32 (19]. Importantly, our data in Figure 1c,d demonstrated that depletion was limited to the CD4^+^CTLA4^+^Foxp3^+^ subset. Importantly, no depletion was observed in any subsets of spleen T cells, including the CD4^+^CTLA4^+^Foxp3^+^ subsets, as shown in Figure 1d,e. The selective depletion of tumor-infiltrating CD4^+^CTLA4^+^Foxp3^+^ correlates with their higher levels of cell surface CTLA-4, as shown in Figure 1a,b,d.

### 3.2. Strategy to Rank Responsiveness of Human Cancer to anti-CTLA-4-Based Immunotherapy

Our strategy, as diagrammed in Supplementary Materials Figure S1, involves two steps. First, we mined the CTLA-4 response database generated by Van Allen et al. [33] to identify four features associated with clinical responders to the anti-CTLA-4 antibody, Ipilimumab, as detailed below. A fifth feature, the ADCC activity, was added to rank therapeutic responses to new generation of ADCC-dependent anti-CTLA-4 antibodies. Second, we analyzed the TCGA RNAseq and DNAseq data from 7279 independent cancer samples belonging to 22 cancer types for components that constituted these five features. The cancer types were ranked based on their median value of the components. Ranking of each feature is obtained from average rankings of 1–3 components. All five features were weighted equally to generate a comprehensive ranking for the 22 types of cancer.

### 3.3. Attribute 1: Ranking Human Cancer Based on CTLA4 Expression

**Figure 2:**
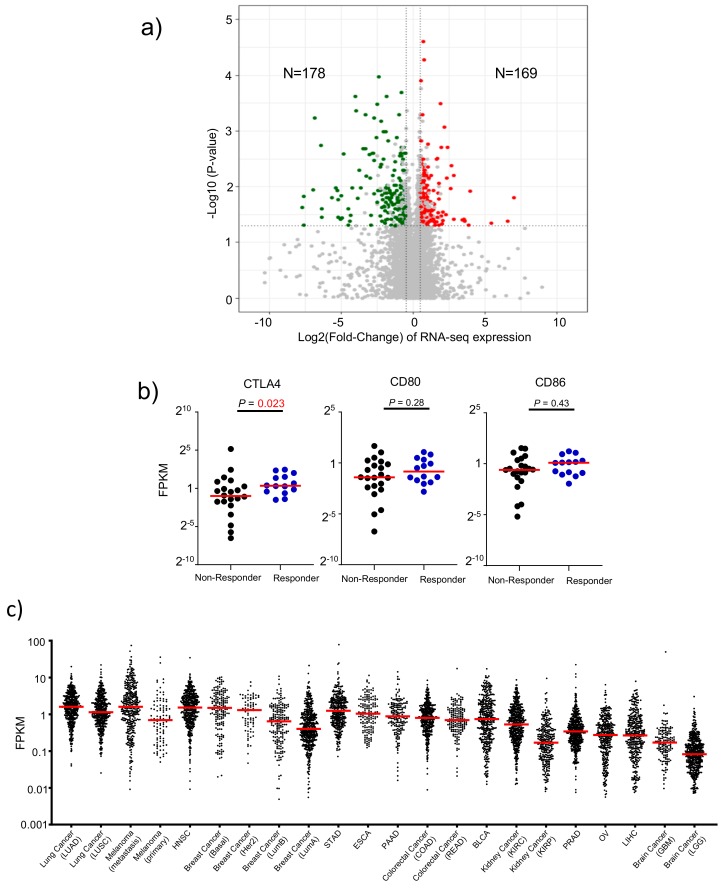
CTLA-4 transcripts associate with therapeutic response to Ipilimumab. (**a**) Volcano plot of mRNA expressions change between anti-CTLA-4 therapy responder and non-responder samples. The x-axis specifies the fold-changes and the y-axis specifies the negative logarithm to the base 10 of the *p*-values. Grey vertical and horizontal dashed lines reflect the filtering criteria. Red and green dots represent genes expressed at significantly higher (*n* = 169) or lower (*n* = 178) levels, respectively. (**b**) The distribution plot shows selected gene expression differences. (**c**) Levels of the *CTLA*4 transcripts among human cancers. The transcript levels of 7279 cancer samples were depicted individually in dot plots. Median values of each cancer type are indicated in horizontal bars.

We mined the anti-CTLA-4 therapeutic response database [33] to identify genes that are significantly associated with responders. As shown in Figure 2a, by comparing transcript levels of all human genes, we found that 169 genes were upregulated significantly while 178 genes were down regulated. Pathway analysis suggests that the upregulated genes are strongly associated with immune responses, while down-regulated genes are not associated with immune responses (Supplementary Materials Figure S1). Among the upregulated genes, CTLA-4 transcript levels are significantly higher among responders to Ipilimumab (Figure 2b). In contrast, those of the CTLA-4 ligands, CD80 and CD86, showed no significant association. These data, together with those in Figure 1, suggest that *CTLA*4 expression is a critical parameter for selective depletion of intratumoral Treg, which prompted us to select *CTLA*4 expression levels in the cancer tissue as a key factor in predicting cancer responsiveness to anti-CTLA-4 antibodies. We therefore ranked 22 human cancer types based on RNAseq data from 7279 independent human cancer samples using the *CTLA*4 expression value. As shown in Figure 2, *CTLA*4 transcripts were found at high levels in most cancer types. Based on median transcript levels, there are about 20-fold differences between the highest and lowest *CTLA*4-expressing cancer types. Among them, non-small cell lung adenocarcinoma (LUAD) ranked at the top, with metastatic lesions of melanoma, basal-type breast cancer, and head and neck squamous cell carcinoma (HNSC) closely following (Table 1). The lowest *CTLA*4-expressing cancer type is low grade glioma of the brain (LGG).

### 3.4. Attribute 2: Ranking ADCC Potential of Anti-CTLA-4 Antibodies in Human Cancer Tissues

**Figure 3:**
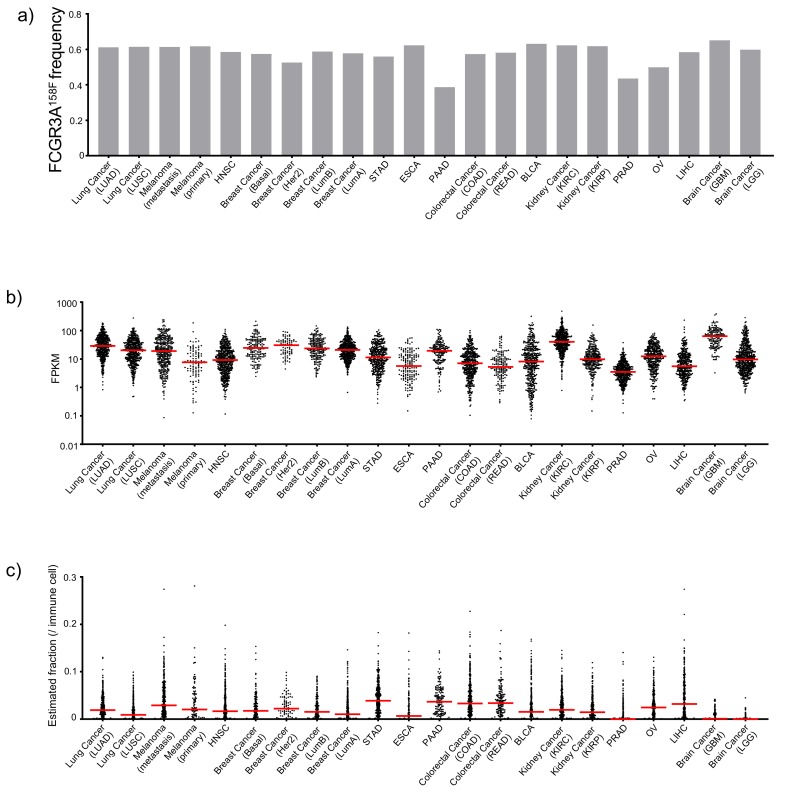
Mining genomic and RNAseq data for ADCC features of 22 cancer types in The Cancer Genomics Atlas (TCGA) database. (**a**) Allele frequency of *FCGR3A*^158*F*^. (**b**) Dot plot showing the *FCGR3A* gene expression. (**c**) Dot plots showing the estimated fraction of Treg cell among tumor-infiltrating leukocytes. Median values of each cancer type are indicated in horizontal bars.

Response to anti-CTLA-4 therapy was reportedly associated with the *FCGR3A*^158*F*^ allele because it encodes a protein with increased IgG Fc binding and thus higher ADCC/ADCP activity [34]. Therefore, we used the158F allele frequency as a contributor of therapeutic responsiveness to ADCC/ADCP-based anti-CTLA-4 therapy. As shown in Figure 3a, most cancer types have similar 158F allele frequency. However, significantly lower 158F frequency was found in pancreatic adenocarcinoma (PAAD), prostate adenocarcinoma (PRAD), and ovarian cancer (OV). Higher levels of *FCGR3A* expression within tumors would be indicative of either the abundance of the *FCGR3A*-expressing cells or higher levels of per cell expression, and both should correlate with ADCC/ADCP activity. We therefore evaluated the levels of the *FCGR3A* transcripts (Figure 3b). This analysis revealed that PRAD, liver hepacellular carcinoma (LIHC) and esophagus carcinoma (ESCA) as the cancer types with the lowest *FCGR3A* gene expression.

Based of tumor-specific Treg depletion in CTLA-4 targeting therapy, the new generation of anti-CTLA-4 therapies should be more effective against tumors with the potential for significant Treg depletion. Therefore, we used the CIBERSORT [24] program to estimate the frequency of tumor-infiltrating Treg. As shown in Figure 3c, most cancer types have significant frequencies of Treg, with highest levels found in stomach adenocarcinoma (STAD), PAAD, colorectal adenocarcinoma (COAD), and LIHC. In contrast, PRAD, glioblastoma multiforme (GBM), and LGG have the lowest Treg fractions.

Since the relative importance of the three attributes to ADCC is unclear, we weighted *FCGR3A*^158*V*^ allele frequcy, *FCGR3A* expression and Treg frequency equally to generate an ADCC score, as shown in Table 1. These analyses assign the highest ADCC ranking to bladder cancer (BLCA), and the lowest ADCC ranking to STAD.

### 3.5. Attribute 3: Transcriptomic Features of Response to Anti-CTLA-4 Therapy

**Figure 4:**
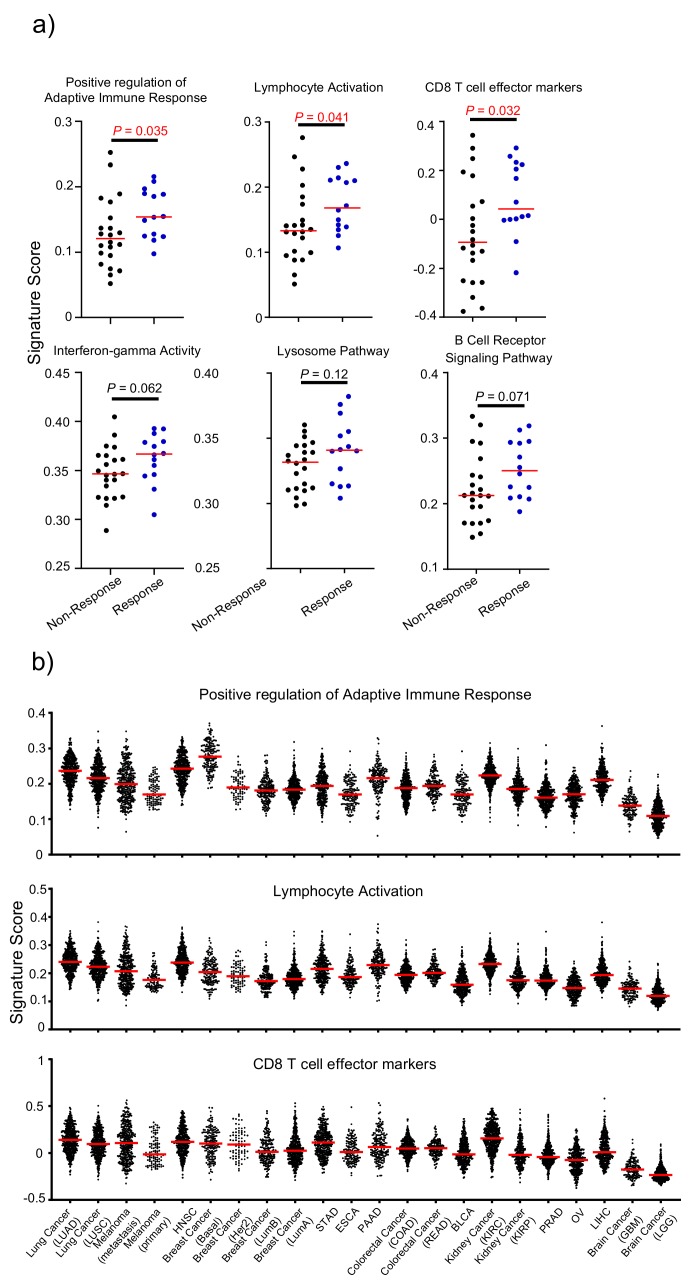
Using immunological signature gene scores validated from the CTLA-4 response database to rank human cancer. (**a**) Distribution plot of the immunological signature gene score change between anti-CTLA-4 therapy responder and non-responder cancer tissues. (**b**) Distribution plot showing three immunological scores of 7279 independent samples from 22 types of human cancer. Those parameters that are significantly associated with CTLA-4 response in (**a**) were chosen to rank the 22 cancer types in (**b**).

To identify a transcriptomic signature for clinical responsiveness of the anti-CTLA-4 currently used in clinic, ipilimumab, we compared immunological signature scores from cancer transcriptome data using single sample gene set enrichment analysis (ssGSEA) with six defined immunological gene sets (Figure 4a). Three of these (“Positive regulation of Adaptive Immune Response”, “Lymphocyte Activation”, and “CD8 T cell effector markers”) were significantly up-regulated in pretreatment samples from anti-CTLA-4 responders. Therefore, we compared 22 cancer types for these three signatures and the average ranking is presented in Table 1. As shown in Figure 4b and Table 1, the strongest signature gene signal in adaptive T cell responses are found in LUAD, HNSC, and kidney renal clear cell carcinoma (KIRC), while the lowest signal was found in GBM and LGG.

### 3.6. Attribute 4: Infiltrating Immune Cell Fraction-Based Ranking

**Figure 5:**
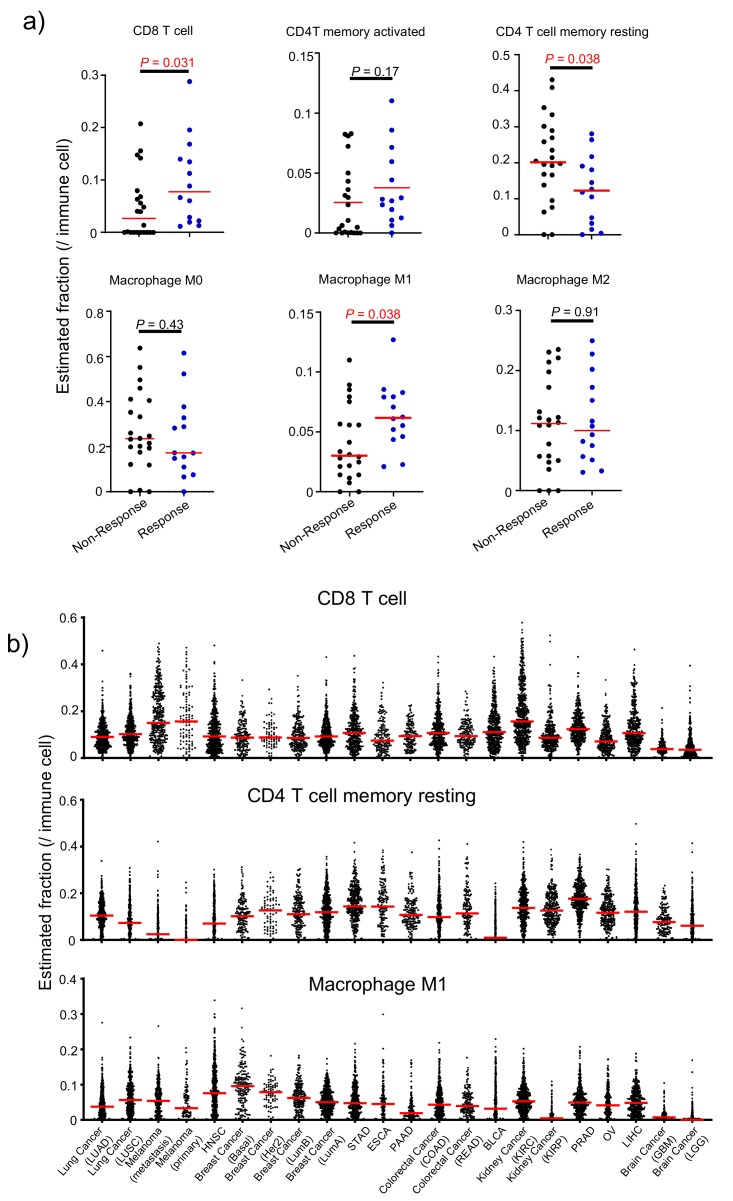
Estimated immune cell fraction varied across cancers from TCGA. (**a**) Distribution plot of estimated immune cell fraction among anti-CTLA-4 therapy responders and non-responders. Those that show significant association were used to rank human cancers. (**b**) Distribution plot shows selected estimated immune cell fractions across 22 cancer types from TCGA. Those parameters that are significantly associated with CTLA-4 response in (**a**) were chosen to rank the 22 cancer types in (**b**).

To investigate whether some specific subsets of leukocytes affects anti-CTLA-4 therapy response rates, we used the CIBERSORT algorithm [27] to estimate the fraction of each leukocyte subset in the tumor microenvironment. Among the major tumor-infiltrating immune effector cells that can be identified by CIBERSORT, including CD8 T cell, CD4T memory activated, CD4 T cell memory resting, macrophage M0, macrophage M1, and macrophage M2, the CD8 T cell and macrophage M1 fractions positively correlate with responsiveness to anti-CTLA-4, while the CD4 T cell memory resting fraction shows a negative correlation (Figure 5a). Other cell types, including activated CD4 memory cells, macrophage M0 and M2 show no significant association with responsiveness to Ipilimumab. We therefore ranked the 22 cancer types based on the frequency of CD8 T cells, macrophage M1 and inversely resting memory CD4 T cells, as shown in Figure 5b. The weighted ranking is summarized in Table 1. This analysis gave metatstatic (SKCM-TM) and primary (SKCM-TP) melanoma top rankings for response to Ipilimumab, and kidney renal papillary cell carcinoma (KIRP) the bottom ranking.

### 3.7. Attribute 5: Tumor Mutation Burden and Neoantigen Counts

It has been reported that tumor mutation burden affects therapeutic response to Ipilimumab [33,35]. Consistent with this notion, the non-silent mutation rate and neoantigen burden were significantly higher in anti-CTLA-4 responder samples when compared with nonresponder samples (Supplementary Materials Figure S2A). The increased mutation rates lead to an expanded pool of potential neo-tumor antigens that also correlate with responsiveness to Ipilimumab (Supplementary Materials Figure S2A). Therefore, we ranked the 22 cancer types based on both mutation burden and neoantigens. As shown in Supplementary Materials Figure S2B and Table 1, SKCM-TM, LUAD, and LUSC (lung squarmous cell carcinoma) are ranked as the top 3 based on mutation burden and neoantigen counts.

### 3.8. A Comprehensive Index to Rank Cancer Responsiveness

Our analyses of five attributes of responsiveness to anti-CTLA-4 therapy allowed us to rank the 22 major human cancers based on individual attributes (Table 1). To generate a comprehensive ranking, we weighted each attribute equally and produced a comprehensive index to rank major cancer types. As shown in Table 1, SKCM-TP, LUAD, HNSC, and LUSC are ranked as the top 4 cancers for potential responsiveness to anti-CTLA-4 therapy. These cancer types have reached the top ranking for different reasons. SKCM-TM, LUSC, and LUAD ranked high based on mutation/Neoantigen burden. SKCM-TM, LUAD, and HNSC ranked high based on CTLA-4 expression. SKCM-TM also ranked first in immune cell infiltration pattern, while LUAD and HNSC ranked high on gene signature. These data emphasize the importance of combining different attributes to rank potential cancer response to anti-CTLA-4 mAbs.

### 3.9. Predicting Selectivity of Treg Depletion in Human Lung Cancer Tissues

**Figure 6:**
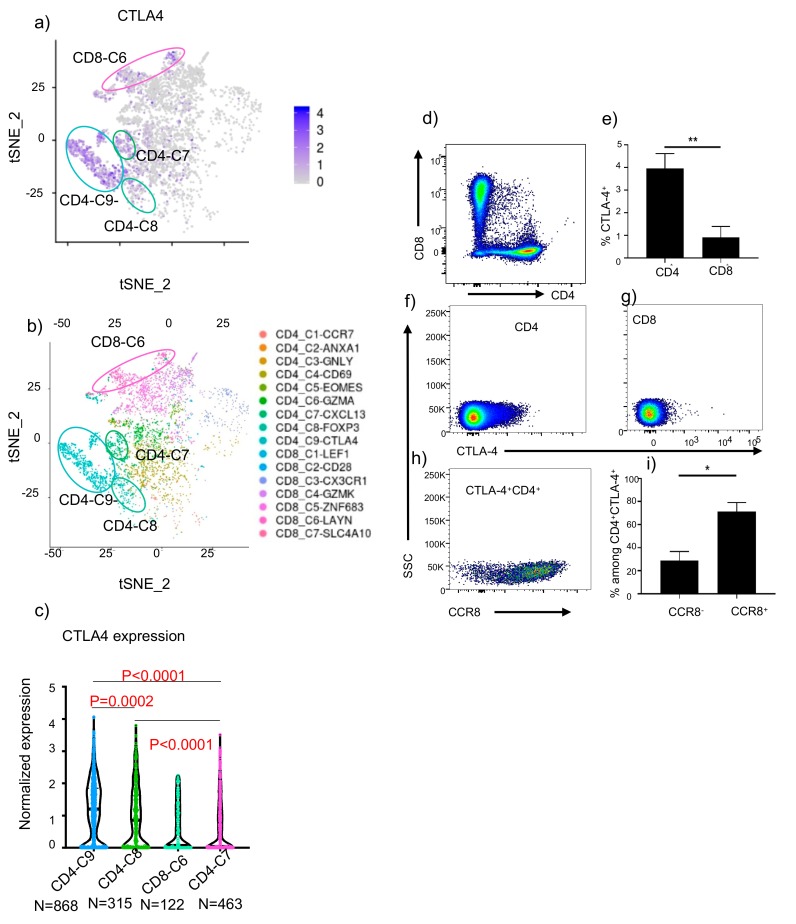
Predicting selectivity of anti-CTLA-4 mAb-mediated Treg depletion in human NSCLC. (**a**) The *CTLA*4 transcript levels among tumor-infiltrating T lymphocytes, using data from the database by Guo et al. [36] (**b**) *CTLA*4 expression among human T lymphocytes classified by Guo et al. [36] (**c**) Violin plot showing the single cell expression pattern of the *CTLA*4 across four indicated clusters. Statistical significance was determined by the One-way ANOVA. (**d**). CD4/CD8 subsets among tumor-infiltrating cells in NSCLC samples. (**e**–**g**) Expression of CTLA-4 among CD4 and CD8 cells. Summary data from 9 cases (**e**) and representative profiles (**f**–**g**) from 1/9 cases are presented. (**h**) Representative profiles of CCR8 and CTLA-4 distribution among tumor infiltrating CD4 T cells. (**i**) Summary data showing the distribution of CCR8^+^ vs. CCR8^−^ cells among CTLA-4-expressing cells.

The most surprising outcome of the above analyses is the predicted high responsiveness of NSCLC, with LUAD ranked No. 2 and LUSC ranked No.4, both below SKCM-TM but well above the SKCM-TP. Since these data may support testing new generation of anti-CTLA-4 antibodies in clinical trials, we decided to perform an in-depth analysis of tumor-infiltrating T cells in human to predict selectivity of anti-CTLA-4 antibody-induced Treg depletion. We first re-analyzed a published dataset of scRNAseq analysis from NSCLC [36] and mapped *CTLA*4 expression among 16 subsets of T cells identified among them. As shown in Figure 6a, of the 16 subsets, the *CTLA*4 transcripts were identified mainly in 4 subsets, which were classified C9, C6, and C7 of CD4 T cells and C8 of CD8 T cells (Figure 6b). We then used the violin plot to show CTLA-4 transcript levels of individual cells in each subset. As shown in Figure 6c, CD4-C9, which are the activated Tregs, expressed the highest levels of CTLA-4 and are the most likely target of anti-CTLA-4 antibodies. CD4-C7, which was called exhausted CD4 T cells, had lower but still significant levels of CTLA-4 but no FOXP3. The other two subsets expressed levels that was far lower than CD4-C9, and thus unlikely to be susceptible. Based on our data in Figure 1, despite expressing significant levels of CTLA-4, the % of FOXP3^−^ CD4 cells are unaffected by anti-CTLA-4 antibodies when tested in an animal model. If this can be extrapolated to human cancer, then it is likely that depletion of CTLA-4-expressing cells will be limited to activated Treg in human cancer.

To confirm distribution of cell-surface CTLA-4 in tumor-infiltrating cells in NSCLC, we analyzed 9 independent fresh NSCLC cancer tissues. The demography of the patients are listed in Supplementary Materials Table S1. As shown in Figure 6d–g, most CTLA-4 expressing cells belong to CD4 subsets. Previous studies have shown that activated Treg in cancer express the CCR8 marker [37]. To test if CTLA-4 levels in tumor-infiltrating cells are higher among activated Treg, we analyzed CTLA-4^+^ cells for their cell surface CCR8. As shown in Figure 6h,i, the overwhelming majority of CTLA-4^+^ cells express CCR8. Furthermore, our re-analysis of the data from Guo et al. [36] showed that CCR8 was selectively expressed on activated Treg in lung cancer tissues (Supplementary Materials Figure S3) and thus serves as a marker for activated Treg. Since this population also expressed the highest levels of CTLA-4 (Figure 6c), it is liely that ADCC-competent anti-CTLA-4 would be highly selective for activated Treg in NSCLC.

## 4. Discussion

Since immunotherapy trials are organized based on cancer types, ranking their potential responsiveness to an immunotherapeutic would be extremely valuable for the selection of clinical indications. This is especially true for CTLA-4-targeting immunotherapy as the best response rates, found in melanoma patients, are about 20%. With the recent understanding on the critical significance of Treg depletion by ADCC/ADCP, and the effort to improve ADCC/ADCP activities of the new antibodies, a timely issue is which cancer types are most suitable for testing the new generation of anti-CTLA-4 antibodies. Here we took a comprehensive approach to rank 22 major cancer types for their responsiveness to anti-CTLA-4 antibodies.

We take advantage of existing RNAseq and DNAseq databases of responders and nonresponders to the anti-CTLA-4 mAb, Ipilimumab [33]. Despite the limited sample size, all components of 4/5 features of the cancer responsiveness ranking have been identified using this database, including *CTLA*4 expression, signature genes, immune cell infiltration, and mutation/Neoantigens.

Although the fifth component, the ADCC/ADCP potential, was not solely identified from this database, others have shown that the *FCGR3A*^158*F*^ allele, which has been shown to be associated with the ADCC activity [34], strongly associated with therapeutic response when combined with tumor mutation burden (TMB) [14]. In addition to the polymorphism, we also added *FCGR3A* expression to our analysis. This is justified based on the clinical experience of the anti-CTLA-4 antibodies, Ipilimumab and Tremelimumab; the former is an IgG1 isotype antibody with strong affinity for FCGRIIIA and has been successful in phase III clinical trials, while the latter is an IgG2 antibody that binds well to FCGRIIA but not FCGRIIIA [34]. In addition, in a mouse model with the human FcR system, it was demonstrated that binding to FCGRIIIA is critical for optimal ADCC of Treg and tumor rejection by anti-CTLA-4 antibodies [34]. In addition to expression and polymorphism of *FCGR3A*, we added Treg frequency to form ADCC/ADCP index. We reasoned that since Treg frequency is a critical prognostic factor in cancer survival [38,39,40,41,42], Treg depletion should have more impact for those cancers with higher Treg infiltration, even though Treg frequency in pre-treatment samples did not show significant correlation with therapeutic response to Ipilimumab, a weak ADCC antibody [19].

It should be cautioned that our primary criteria were based on the database of melanoma patients as we are constrained by the fact that clinical efficacy of monotherapy targeting of CTLA-4 has been proven through phase III clinical trials only for this indication. Therefore, the relative ranking may be subject to modification should further clinical studies show clinical efficacy in other indications where comprehensive data are available.

Obviously, a cancer-type based ranking will necessarily overlook heterogeneity of cancer samples within a cancer type. While high ranking suggests high chance of success if indications are chosen without pre-selecting patients within the cancer type, a low ranking of the cancer type does not suggest all patients of that cancer type should be ineligible for clinical trials of new anti-CTLA-4 antibodies. In fact, we have presented data of individual data points in most figures to highlight the fact there are patients in most cancer types with features that are favorable for anti-CTLA-4 therapy. The five features we used to rank cancer types could also be used to evaluate whether an individual will be indicated for anti-CTLA-4 therapy in a personalized medicine approach.

Metastatic melanoma receiving the highest ranking. The fact that Ipilimumab has been shown to efficacious in treating metastatic melanoma, both as mono-immunotherapy [43] or a combination with anti-PD-1 antibody Nivolumab [43,44] validates the ranking system described herein. It is worth noting that 10 other cancer types rank above primary melanoma, which responded to Ipilimumab as an adjuvant therapy [45]. Therefore, it is likely multiple cancer types may well respond to anti-CTLA-4 therapy if appropriate antibodies are employed. In particular, LUAD ranks No. 2, and LUSC ranks No. 4 among the 22 cancer types. Our re-analysis of scRNAseq data of tumor-infiltrating T cells in NSCLC patients suggests that selective Treg depletion is achievable with anti-CTLA-4 antibodies.

While the high ranking suggests NSCLC as a prime candidate for new anti-CTLA-4 antibodies, it is worth considering the fact that multiple randomized trials have failed to show significant improvement in survival of patients receiving Ipilimumab in combination with chemotherapy [8]. Therefore, subsequent studies focused on the dual blockade of CTLA-4 and PD-1/PD-L1 [46]. Recently, two randomized phase III trials (CheckMate 227 and MYSTIC) showed improved activity compared with platinum-based chemotherapy only in selected patients with high tumor mutation burden with ipilimumab-nivolumab [47], while tremelimumab-durvalumab [48] failed to demonstrate any benefit in unselected patients. Therefore, a new generation of anti-CTLA-4 antibodies must show higher anti-tumor activity in order to achieve better outcomes. Moreover, due to high toxicity, high rates of patient drop out have been observed in the Ipilimumab group. In addition to the safety concern, high patient dropout and cross-over in the trials also impede data interpretation. In this context, we recently showed that increasing pH sensitivity of anti-CTLA-4 antibodies improves both therapeutic effect and safety when compared directly with Ipilimumab [19]. Since Treg has been implicated in pathogenesis and prognosis of multiple cancer types [35,36,37,38,39], it is anticipated that anti-CTLA-4 antibodies that effectively and selectively deplete Treg in tumor microenvironment would have a broad impact in cancer immunotherapy.

## 5. Conclusions

In summary, our in silico and experimental analyses suggest that non-small cell lung carcinoma will likely respond to a new generation of anti-CTLA-4 monoclonal antibodies. Our approach provides an objective ranking of the sensitivity of human cancers to anti-CTLA-4 antibodies. The comprehensive ranking provides a roadmap for the clinical development of the next generation of anti-CTLA-4 antibodies.

## Figures and Tables

**Table 1:** Ranking human cancer for their responsiveness to anti-CTLA-4 therapy.

Order	Type	Rank Number					
		ADCC Feature	CTLA4 Expression	Signature Genes Score	Immune Cell Infiltration	Mutational Burden	Sum
1	SKCM-TM	13.7	2	6.7	4.0	1.0	27.3
2	LUAD	12.7	1	1.3	13.0	3.5	31.5
3	HNSC	10.7	3	2.7	6.7	12.0	35.0
4	LUSC	12.7	8	5.7	6.7	4.5	37.5
5	BRCA-Basal	8.0	4	5.3	8.7	12.0	38.0
6	BRCA-Her2	10.0	5	10.3	11.7	12.5	49.5
7	KIRC	11.0	15	2.7	9.3	13.0	51.0
8	COAD	10.3	10	11.0	9.0	12.0	52.3
9	PAAD	13.0	9	6.3	13.7	12.0	54.0
10	BLCA	5.3	11	18.3	8.3	13.0	56.0
11	STAD	21.3	6	6.7	12.7	11.0	57.7
12	SKCM-TP	15.0	12	16.0	6.3	10.5	59.8
13	READ	13.3	13	9.3	12.7	12.0	60.3
14	BRCA_LumB	8.0	14	15.3	10.7	12.5	60.5
15	BRCA_LumA	7.3	16	13.3	12.3	12.5	61.5
16	ESCA	10.7	7	15.0	17.0	13.0	62.7
17	LIHC	11.3	19	11.0	11.3	13.0	65.7
18	PRAD	13.0	17	18.7	11.3	12.5	72.5
19	OV	12.7	18	18.7	16.0	12.5	77.8
20	KIRP	11.0	21	15.7	18.3	13.0	79.0
21	GBM	10.7	20	21.0	16.0	13.0	80.7
22	LGG	11.0	22	22.0	16.0	11.5	82.5

## Supplementary Materials

The following are available online at https://www.mdpi.com/2072-6694/12/2/284/s1, Figure S1: Diagram of the strategy to rank human cancer for their responsiveness to anti-CTLA-4 antibody treatment., Figure S2: Mutational burden varied across cancers from TCGA. Table S1: Patient information, related to Figure 6.

## Abbreviations

TCGA	The Cancer Genome Atlas
GSEA	Gene-set Enrichment Analysis
ADCC	Antibody Dependent Cell-mediated Cytotoxicity/phagocytosis
ADCP	Antibody Dependent Cell-mediated Phagocytosis
NSCLC	Non Small Cell Lung Cancer
TMB	Tumor Mutation Burden
