# Supplementary material for: Mechanism- and Immune Landscape-Based Ranking of Therapeutic Responsiveness of 22 Major Human Cancers to Next Generation Anti-CTLA-4 Antibodies
Source: Cancers (Basel). 2020 Jan 24;12(2):284. doi: 10.3390/cancers12020284 (PMC7073233; doi:10.3390/cancers12020284)
Supplement: Supplementary file 1 [file cancers-12-00284-s001.pdf]

Supplemental information

Supplemental Table S1. Patient information, related to Figure 6.

Patient	Sex	Age	Stage	NSCLC Type
P1	Male	66	IIIA	Adenocarcinoma
P2	Female	69	IB	Squamous cell carcinoma
P3	Male	67	IA3	Adenocarcinoma
P4	Female	62	IA3	Adenocarcinoma
P5	Male	59	IB	Squamous cell carcinoma
P6	Male	50	IA	Adenocarcinoma
P7	Male	66	IIIA	Squamous cell carcinoma
P8	Female	59	IIIA	Adenocarcinoma
P9	Male	56	IIB	Squamous cell carcinoma

Supplementary Figure 1

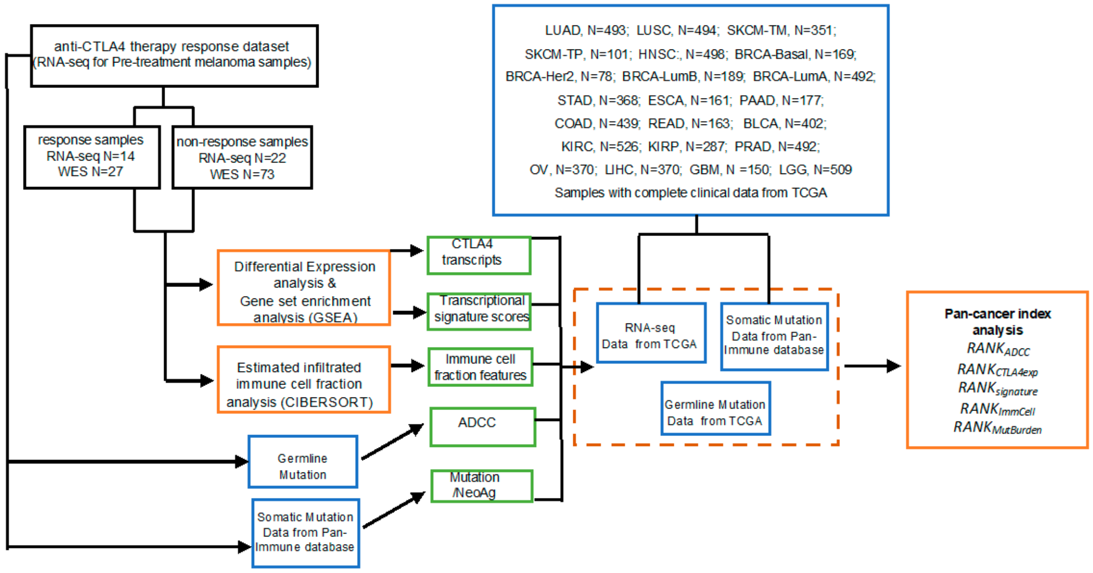

Supplemental Figure S1. Diagram of the strategy to rank human cancer for their responsiveness to anti-CTLA-4 antibody treatment. We first identified attributes of therapeutic response of anti-CTLA-4 antibodies based on a CTLA-4 response database [33], taking into consideration the newly identified mechanism of action by ant-CTLA-4 antibodies and analysis performed by another group on *FCGR3A* polymorphism and responsiveness to Ipilimumab. The human cancer types were ranked based on the median value of each attributes. The rankings of the five attributes were then weighted to yield a final ranking.

Supplementary Figure 2

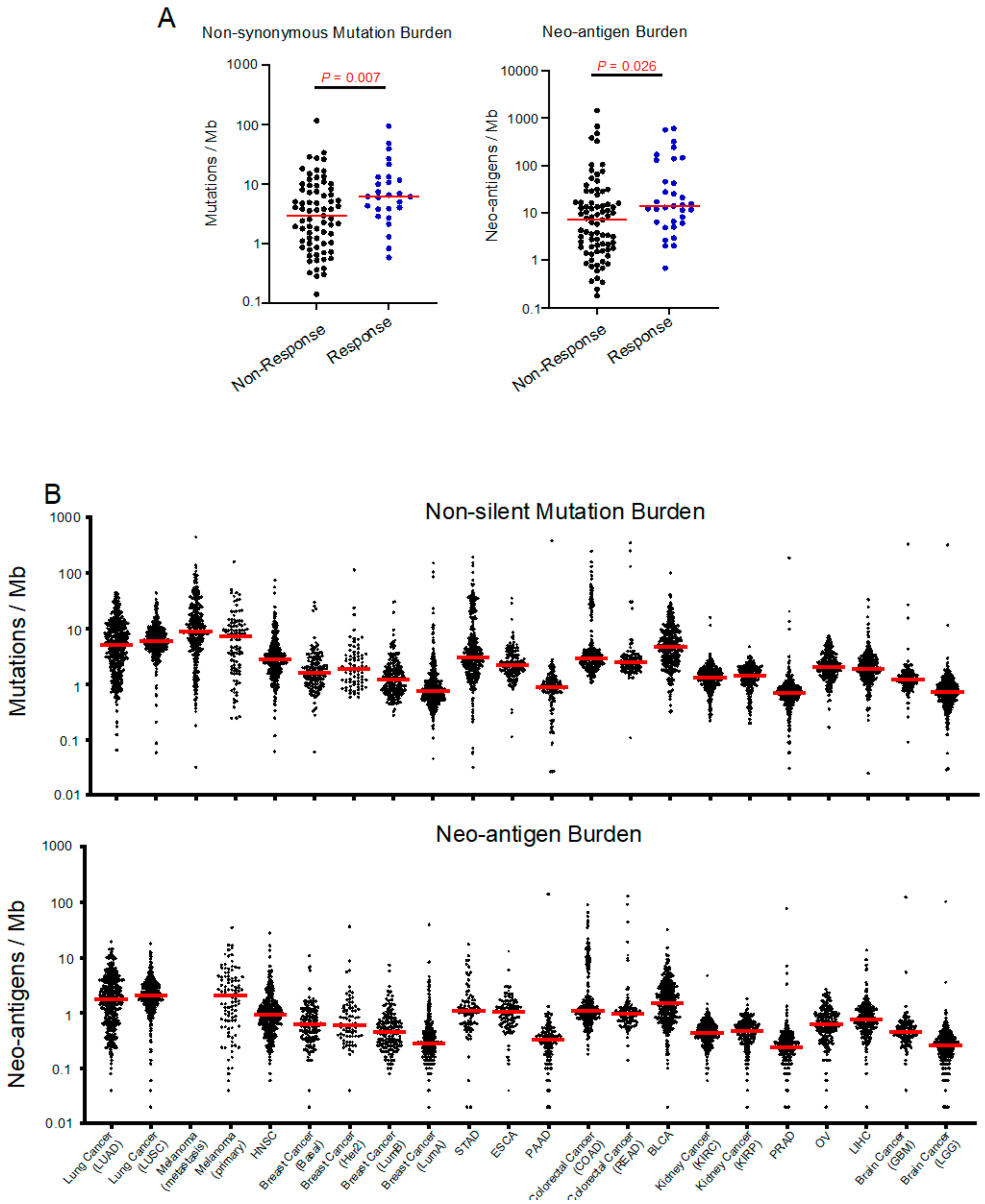

Supplemental Figure S2. Mutational burden varied across cancers from TCGA. (A) Distribution plot of non-synonymous mutation count and neo-antigen count among anti CTLA-4 therapy responder and non-responder samples. (B) Distribution plot shows mutational burden and neoantigen counts across 21 cancer types of human cancer. The neoantigen counts for metastatic melanoma was unavailable.

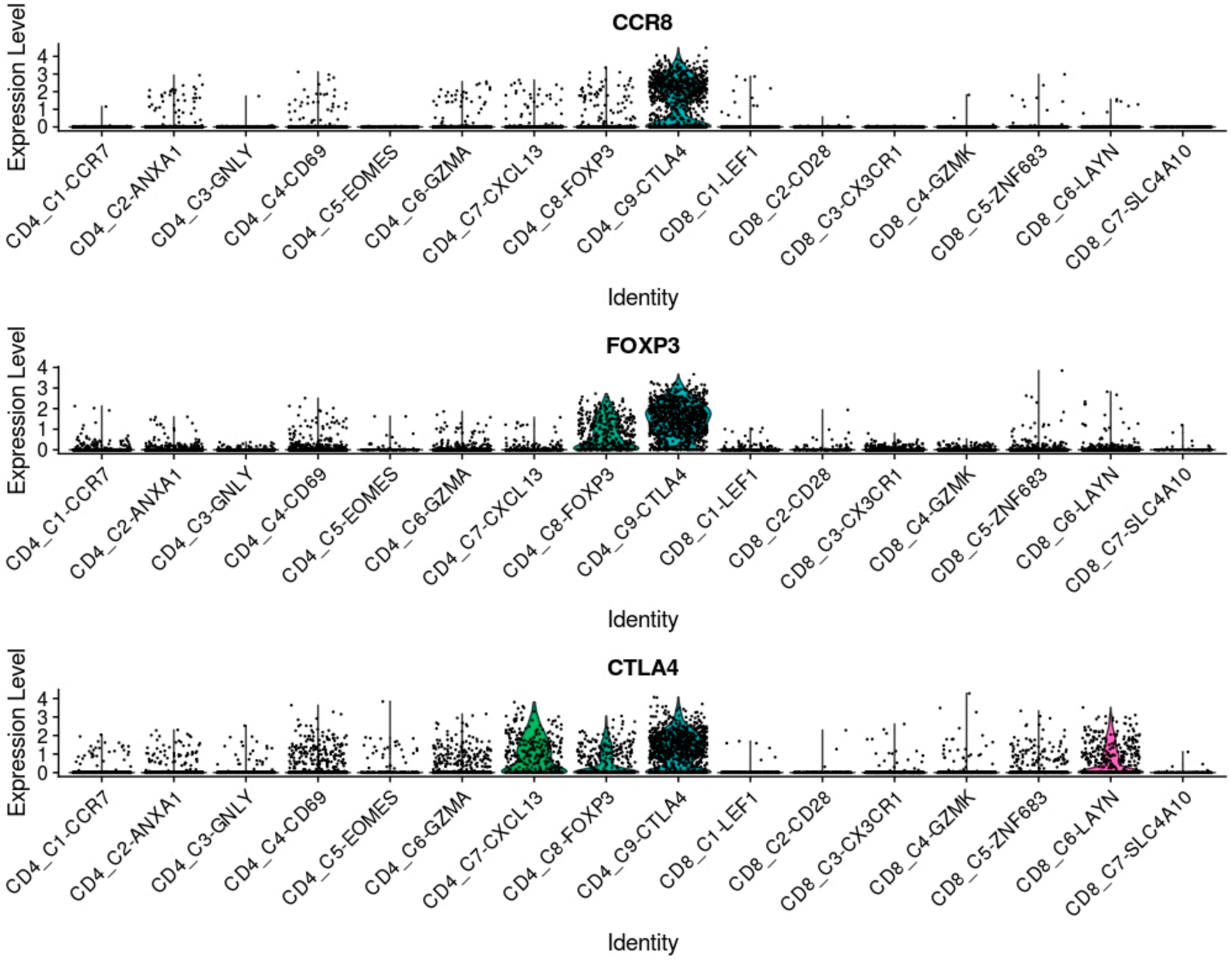

Supplemental Figure S3. **CCR8 is a marker for activated Treg that express high levels of CTLA-4 and FOXP3**. Data shown are re-analysis of scRNAseq data from Guo et al [36]. Related to Figure 6.
